# Piezoelectric energy harvester with double cantilever beam undergoing coupled bending-torsion vibrations by width-splitting method

**DOI:** 10.1038/s41598-021-04476-1

**Published:** 2022-01-12

**Authors:** Jiawen Song, Guihong Sun, Xin Zeng, Xiangwen Li, Quan Bai, Xuejun Zheng

**Affiliations:** 1 School of Mechanical Engineering, Engineering Research Center of Complex Tracks Processing Technology and Equipment of MoE, Key Laboratory of Welding Robot and Application Technology of Hunan Province, Xiangtan University, Xiangtan, 411105 People’s Republic of China; 2 Key Xiangtan Hongda Vacuum Technology Co., Ltd., Xiangtan, 411104 People’s Republic of China; 3 Wasion Electric Co., Ltd., Xiangtan University, Xiangtan, 411199 People’s Republic of China

**Keywords:** Engineering, Mechanical engineering

## Abstract

We propose piezoelectric energy harvester (PEH) with double-cantilever-beam (DCB) undergoing coupled bending-torsion vibrations by combining width-splitting method and asymmetric mass, in order that more ambient energy could be harvested from environmental vibration with multiple-frequency excitation. The geometrical dimensions are optimized for PEHDCB, when the maximum of output peak voltages U_p-max_ and resonance frequency difference (Δ*f*_0_) between the first and second modes are chosen as optimization objectives based on orthogonal test method. The energy harvesting efficiency is evaluated by the proportion of half-power bandwidth and quality factor, and the experimental and simulation results are compared to verify reliability. The U_p-max1_ and P_p-max1_ are increased 25.2% and 57.3% for PEHDCB under the multi-frequency excitation, when the split-width method is applied into PEH with single-cantilever-beam (SCB) undergoing coupled bending-torsion vibrations. The deviations of U_p-max1_ and *f*_0_ are at the ranges of 4.9–14.2% and 2.2–2.5% for PEHDCB under the different mass ratios, and the measurement reliability is acceptable considering incomplete clamping, damping and inevitable assembly effects. The energy harvesting efficiency of PEHDCB presented is much higher than that of the conventional PEHSCB from environmental vibration with multiple-frequency excitation.

## Introduction

In recent years, harvesting ambient energy using piezoelectric effect is considered to be an important energy conversion technology applicable to future industries along with photovoltaics^1^, thermoelectrics^2^, radio frequencies^3^, and triboelectric technology^4^. Vibration-based piezoelectric energy harvester is a potential candidate to replace existing power sources such as the batteries which have a limited energy storage capacity and lifetime for some applications^5^. Based on Taguchi orthogonal method, the variation in the excitation frequency, the thickness of piezoelectric layers and the acceleration of the bimorph piezoelectric energy harvester (PEH) were optimized by maximum output peak voltage (U_p-max_) and signal-to-noise ratio as the optimization objectives^6^, and the ultimate goal is that one could obtain the U_p-max_ with lesser trials^7^. Most of PEHs with the different beam shapes, such as the rectangles^8–11^ and triangles^12,13^ have been focused on the energy harvesting characteristics from bending vibration, and the main problem is the significant dropping of output power when the excitation frequency slightly away from the natural frequency of the harvesters. The durability of PEH is importance to sustainable power wireless sensors, and some researchers have focused on the fatigue analysis for PEH under different excitation cycles^14^ and levels of base excitation^15^.

PEH can effectively harvest electric power from torsional vibration by attaching a simple cantilever beam structure on the top surface of a rotating shaft generate^16^. A unimorph cantilever beam undergoing bending-torsion vibrations has been proposed by asymmetry increasing under a transverse harmonic base excitation to narrow the resonance frequency difference (Δ*f*_0_) between first and second modes, therefore it allows the harvesting of electrical power from multiple-frequency excitation^17^. The novel asymmetric vortex-induced piezoelectric harvester for capturing wind energy at low wind speed is of lower natural frequency and smaller electromechanical coefficient than those of the conventional vortex-induced piezoelectric energy harvester^18^. The effect of width-splitting on the harvested power was theoretically and experimentally investigated for a fixed dimension piezoelectric materials of PEH undergoing bending vibration, and the width-splitting method could be used to increase the harvested voltage^19^ and power^20^ over a wider range of excitation frequencies. The single piezoelectric beam with the similar total width was folded equally and then split a given dimension of piezoelectric materials with the predefined dimensions into the array of smaller-width beams, and there is a substantial increase in harvested power^21^. There are many reports on the evaluation methods of half-power bandwidth and quality factor for PEHs undergoing bending vibration^19,21^, however relatively few researches are focus on the evaluation method for PEH undergoing coupled bending-torsion vibrations. The best performance occurs at a single or very narrow frequency range for most resonating harvesters, and then a critical issue is how to adjust the resonance frequency (*f*_0_) flexibility and maximize harvesting power because of environment vibrations composed of some broadband random or multiple-frequency excitation^22,23^. In a word, the width-splitting method could be used to improve energy harvesting performances of PEH undergoing bending vibration, however one could not know whether it is applicable to harvest more ambient energy from multiple-frequency excitation by using PEH undergoing coupled bending-torsion vibrations.

In this paper, PEH with single-cantilever-beam (SCB) is equally split into PEH with double-cantilever-beam (DCB) in order to harvest ambient energy from multiple-frequency excitation, which is verified comparing with the experimental and simulation results of the U_p-max_ and Δ*f*_0_. After the geometrical dimensions including the split gap *d,* primary beam *L* and substrate thickness *t*_s_ were determined by using U_p-max_ and Δ*f*_0_ combination in multi-factors analysis based on orthogonal test method, we had fabricated PEHSCB and PEHDCB undergoing coupled bending-torsion vibrations. The mechanical vibration experiments were performed by shaker to measure U_p_ vs frequency (U_p_-*f*) curves of PEHs undergoing coupled bending-torsion vibrations, and the half-power bandwidth proportion and materials mechanical quality factors are used to evaluate the harvesting efficiency from the multiple-frequency excitation. We expect that the research could significantly offer some useful guidelines to design high-performance PEH, which could harvest more ambient energy under multiple-frequency excitation.

## Design and simulation

### Construction of PEHDCB split from PEHSCB

**Figure 1:**
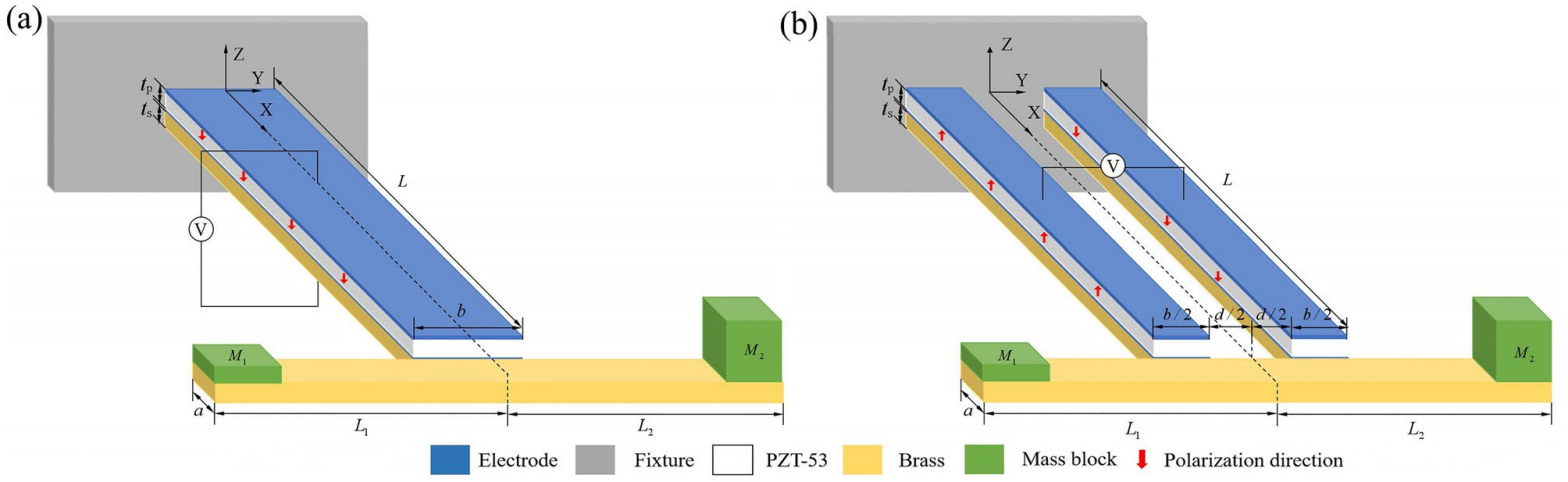
Schematics of PEH (**a**) SCB and (**b**) DCB undergoing coupled bending-torsion vibrations.

The single/double T-shaped brass shims and commercial piezoelectric ceramic PZT bulks were provided by Wuxi Hui Feng Electronics Co. Ltd, and the material properties parameters^24–26^ are listed in Table 1. PZT element was formed of PZT wafer, top and bottom electrodes, before PEHSCB was designed by bonding the elements on the primary beam via conductive adhesive, as shown in Fig. 1a. PEHDCB is constructed by splitting equally the primary beam of PEHSCB, as shown in Fig. 1b, and the split gap *d* determined by the width-splitting method^19,21^ is listed in Table 2.

**Table 1:** The materials properties.

**Brass**			
Young’s modulus (GPa)	100	Poisson’s ratio	0.3
Density (kg/m^3^)	9000	Yield strength (MPa)	200
**PZT-53**			
Density (kg/m^3^)	7750	Yield strength (MPa)	60
Mechanical quality factor Q_m_	65	Mechanical damping ratio ζ_m_	0.007
Elastic compliance constant (10^–12^ m^2^/N)		$s_{{{11}}}^{{\text{E}}}$ = 15 $s_{{{33}}}^{{\text{D}}}$ = 8.8 $s_{{{55}}}^{{\text{D}}}$ = 22	
Piezoelectric strain constant (10^–12^ C/N)		${\text{d}}_{15}$ = 1050 ${\text{d}}_{33}$ = 590 ${\text{d}}_{31}$ = − 270

**Table 2:** The geometrical dimensions.

*d* (mm)	*L* (mm)	*t*_s_ (mm)	m*	*t*_p_ (mm)	*L*_1_/*L*_2_ (mm)	*a* (mm)	*b* (mm)
2–6	26–30	0.4–0.8	0–19	0.4	22	6	12

There are two variable mass blocks *M*_2_ and *M*_1_ placed at tip of the crossbeam, and *M*_2_/*M*_1_ is defined as the mass ratio m^*^ and when the mass ratio changes, the mass sum of mass blocks remains unchanged at both of the beam ends. The geometrical dimensions of PEHSCB include the length *L*, width *b*, thickness *t*_s_, piezoelectric ceramic thickness *t*_p_ for the primary beam and the length *L*_1_, *L*_2_, width *a*, mass ratio m* for the crossbeam, and all of them are listed in Table 2*.* The polarization direction of piezoelectric ceramic PZT-53 bulk is marked by the red arrows along Z-axis. Learning from the series connection structure^27^, two PZT elements split from the primary beam of PEHSCB are poled oppositely in the perpendicular to length direction to form the series connection shown in Fig. 1b. In Cartesian coordinate system (X, Y, Z), one ends of the single and double beams are fixed, and the crossbeams are at free end.

### Simulation

#### Modeling on PEHSCB and PEHDCB

The U_p_ and *f*_0_ of PEHs are simulated by using the commercial analysis package ANSYS. The 8-node hexahedral coupled-field element SOLID5 and the 8-node linear structural element SOLID45 are used for piezoelectric materials and non-piezoelectric materials, and piezoelectric circuit element CIRCU94 is used for load resistor. The displacement degrees of freedom are constrained to be zero for fixed end of the primary beam of PEH. The electrode connections are made by using the “couple” commands^28^. As for PEHDCB, the potentials of top surface of substrate layer are coupled with bottom surface of piezoelectric layer, and they are individually coupled for top surfaces of two piezoelectric layers. The potential is constrained to be zero for one top surface of piezoelectric layers, and a load resistor is connected between the top surfaces of two piezoelectric layers, as shown in Fig. 1. The output voltage across load resistor is solved for the potential of top surface of piezoelectric layer, when T-shaped beam is loaded with the inertial acceleration 9.8 m/s^2^.

**Figure 2:**
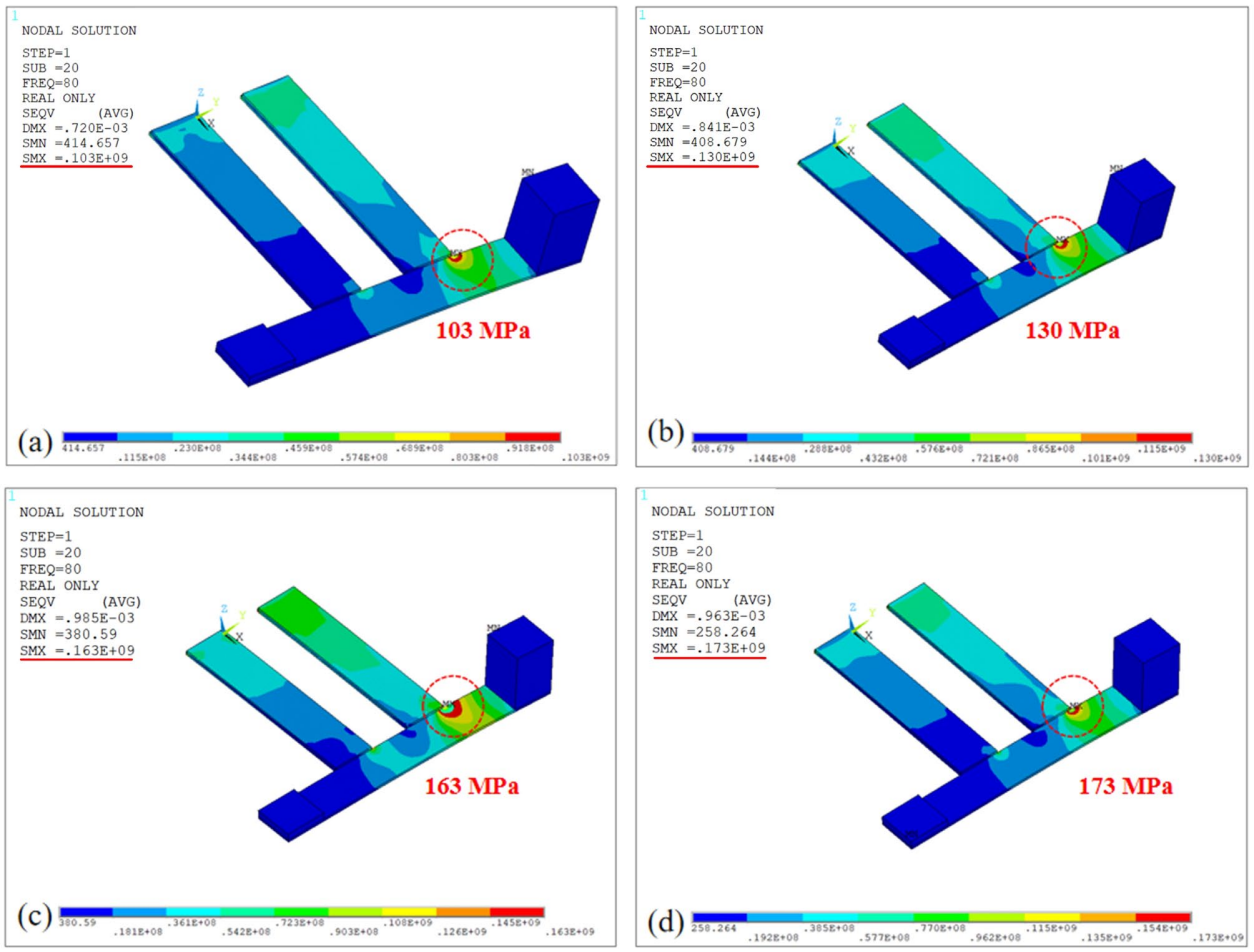
The modal stress diagrams of convergent judge at the mesh densities for PEHDCB (**a**) 0.7, (**b**) 0.6, (**c**) 0.5 and (**d**) 0.4 mm.

In order to obtain a convergent solution by using mesh refinement^29^, the modal stress diagrams at the first mode of PEHDCB are performed by considering the four different mesh densities 0.7, 0.6, 0.5 and 0.4 mm, and they are respectively given as in Fig. 2a,b,c,d. Obviously, the difference of the maximum stress is less than 6% for the last two mesh densities, as shown in Fig. 2c,d, therefore it could be regarded as a convergent solution when mesh density 0.5 mm.

#### The modal stress analysis

**Figure 3:**
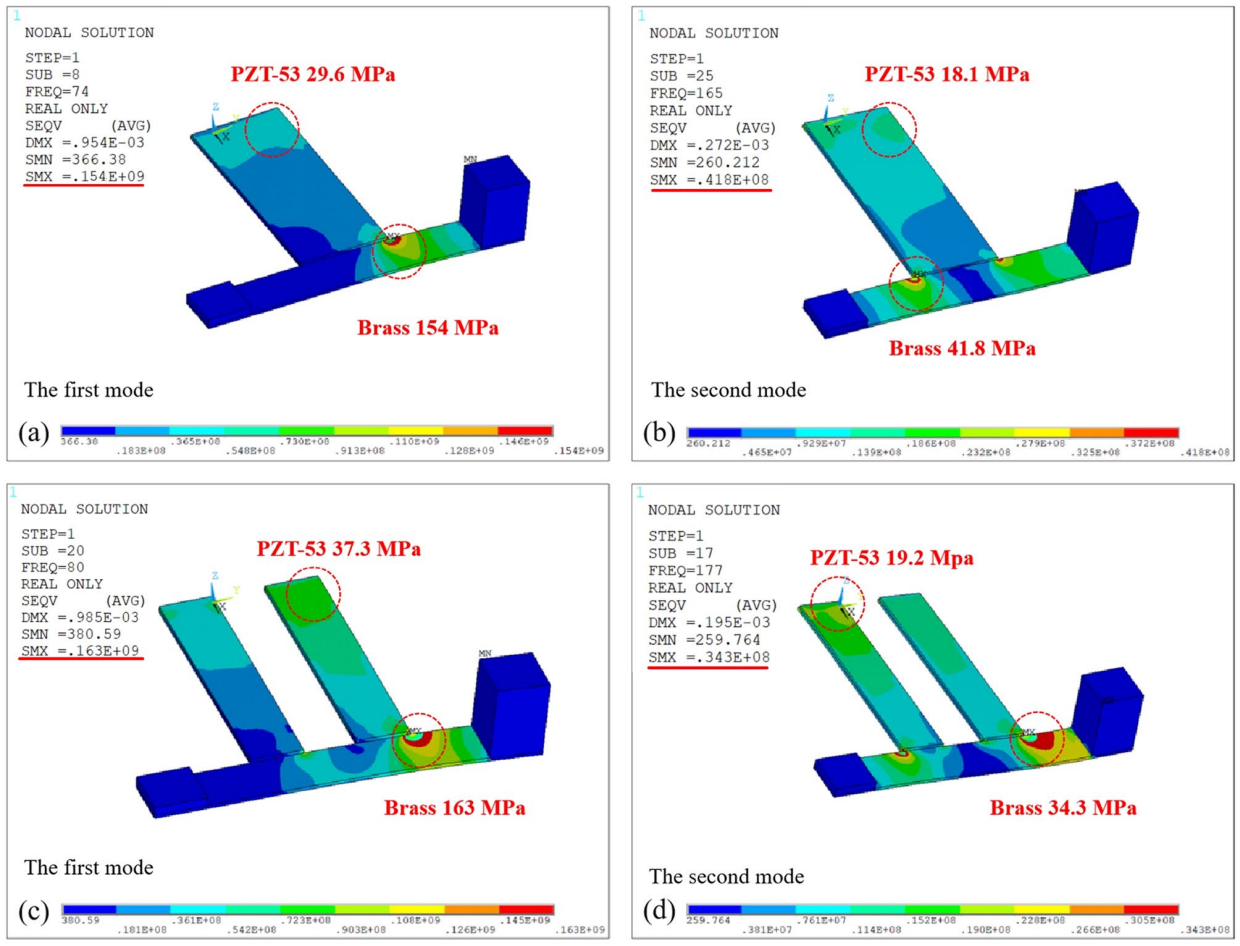
The modal stress diagrams of yield strength (**a,b**) for PEHSCB and (**c**,**d**) for PEHDCB.

In order to satisfy the yield strength of piezoelectric materials and brass^30^, the modal analyses on PEHs with asymmetric mass are performed under the mesh density 0.5 mm, and the modal stress diagrams are presented in Fig. 3a,b for PEHSCB and Fig. 3c,d for PEHDCB. Obviously, the maximum stresses of piezoelectric layer 37.3 MPa and brass layer 163 MPa occur at the first mode for PEHDCB as shown in Fig. 3c, and they are smaller than the yield strengths of 60 MPa and 200 MPa, indicating yield strength reliability.

#### Optimization on asymmetry PEHs with the different mass ratios

**Figure 4:**
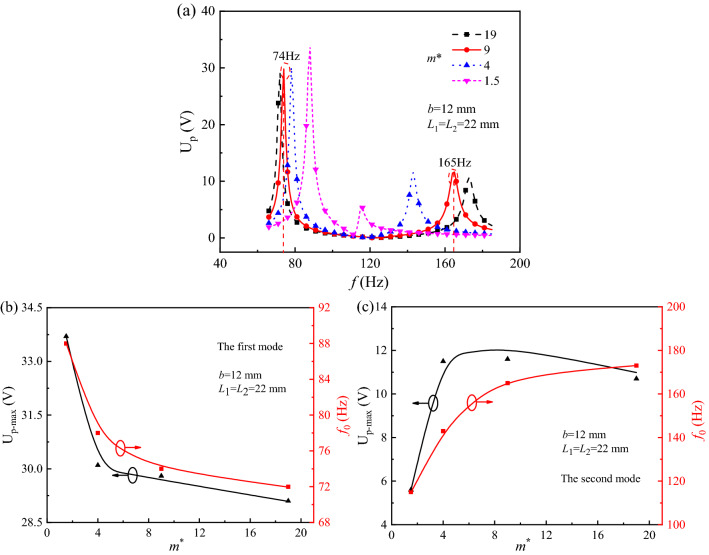
The U_p_-*f* curves (**a**), U_p-max_ and *f*_0_ vs m^*^ curves at the first (**b**) and second (**c**) modes of PEHSCB.

To investigate the effect of m^*^ on the harvesting performance of PEHSCB, the U_p_-*f* curves were simulated under the different mass ratios m^*^ = 19, 9, 4 and 1.5 when *b* = 12 mm, *L*_1_ = *L*_2_ = 22 mm, and they are described as Fig. 4a. With the asymmetry increase of m^*^ from 1.5 to 19, the U_p-max1_ and *f*_01_ at the first mode decrease from 33.6 to 29.0 V and 88 to 72 Hz. On the contrary, the U_p-max2_ and *f*_02_ at the second mode increase from 5.1–11.6 V and 118 to 177 Hz. As for m^*^ > 1, all of the *f*_01_ and *f*_02_ are far lower than the nature frequency 200 Hz and the Δ*f*_0_ is narrowed. Therefore it allows the harvesting of electrical power from the multiple-frequency excitation, and they are agreement with the previous results^17^. Drawing the U_p-max_ and *f*_0_ at the certain m^*^ from Fig. 4a, the U_p-max_ vs m^*^ and *f*_0_ vs m^*^ curves are described as Fig. 4b for the first mode and Fig. 4c for the second mode. Both of the U_p-max1_ and *f*_01_ decrease with the asymmetry increase of m^*^, as shown in Fig. 4b, however the *f*_02_ increases and the U_p-max2_ increases to reach a peak value at m^*^ = 9 and then decreases by contrary, as shown in Fig. 4c. The optimization mass ratio m^*^ is determined as 9 by considering the trade-off between U_p-max_ and *f*_0_, in order that PEHSCB could achieve not only the higher U_p-max_ but also both the lower *f*_01_ and *f*_02_. According to the previous analysis method^31^, we analogously determine the mass ratios m^*^ = 9 to investigate harvest ambient energy of PEHDCB undergoing coupled bending-torsion vibrations from a multiple-frequency excitation as follow.

#### Effect of single-factor on PEHDCB

**Figure 5:**
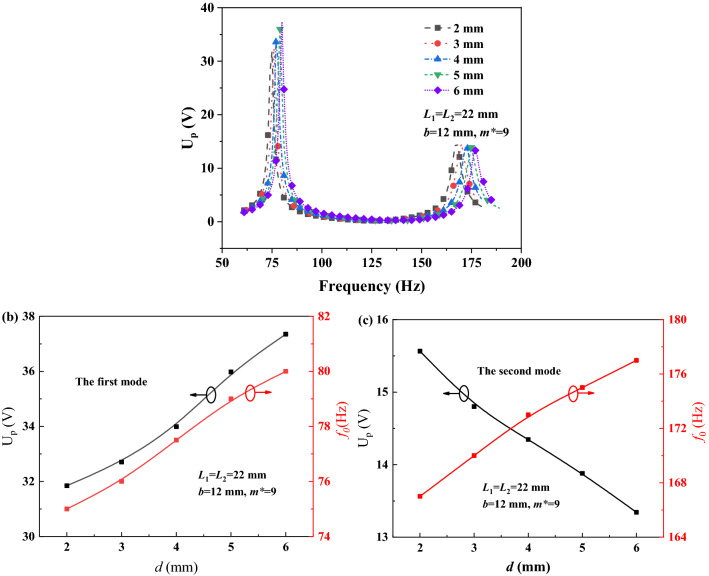
The U_p_-*f* curves (**a**), U_p-max_ and *f*_0_ vs m^*^ curves at the first (**b**) and second (**c**) modes.

To understand the effect of single geometrical parameter on energy harvesting performance of PEHDCB, the U_p_-*f* curves were simulated under the different split gaps *d*, such as 2, 3, 4, 5 and 6 mm, and they are described as Fig. 5a when m^*^ = 9, *b* = 12 mm, *L*_1_ = *L*_2_ = 22 mm. With the *d* increase from 2 to 6 mm, the U_p-max1_, *f*_01_ and *f*_02_ increase from 31.8 to 37.3 V, 75 to 80 Hz and167 to 177 Hz however the U_p-max2_ decreases from 14.6 to 13.3 V. The dependencies of U_p-max_ and *f*_0_ on m^*^ are analogously discussed as shown in Fig. 4b,c, and the U_p-max_ and *f*_0_ vs *d* curves are described as Fig. 5b for the first mode and Fig. 5c for the second mode. With the increase of *d*, both of the U_p-max1_ and *f*_01_ increase monotonously, however the U_p-max2_ decreases and the *f*_02_ increases by contrary. By adjusting the split gap *d*, PEHDCB can harvest energy more evenly at the two first modes, and it is in an agreement with the reported result^32^.

**Figure 6:**
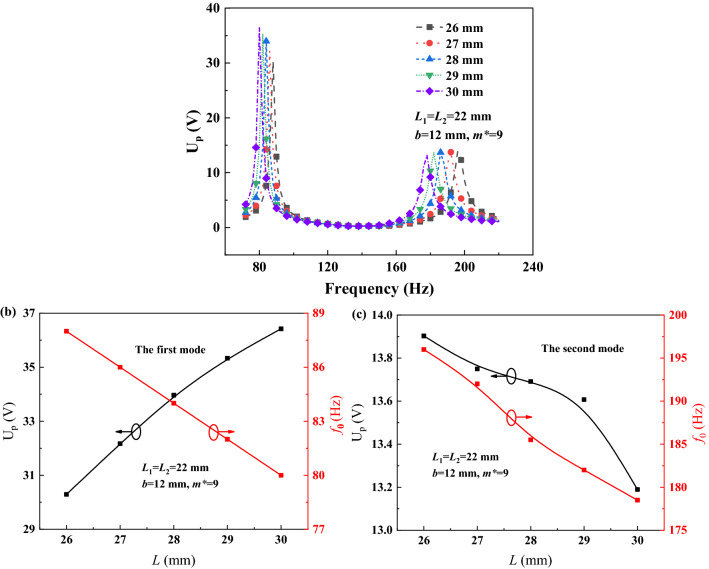
The U_p_-*f* curves (**a**), the U_p-max_ and *f*_0_ vs *L* curves at the first (**b**) and second (**c**) modes.

The U_p_-*f* curves were simulated under the different primary beam lengths *L*, such as 26, 27, 28, 29 and 30 mm, *L*_1_ = *L*_2_ = 22 mm, and they are described as Fig. 6a when m^*^ = 9, *b* = 12 mm. The *d* dependencies of U_p-max_ and *f*_0_ are analogously discussed, as shown in Fig. 5b,c, and the U_p-max_ and *f*_0_ vs *L* curves are described as Fig. 6b for the first mode and Fig. 6c for the second mode. With the increase of *L*, the U_p-max1_ monotonously increases but the U_p-max2_ slightly decreases at the second mode, however both of the *f*_01_ and *f*_02_ decrease. It indicates that the primary beam length can be adjusted by considering the trade-off between the U_p-max_ and *f*_0_, in order that PEHDCB could harvest energy more evenly under the multiple-frequency excitation.

**Figure 7:**
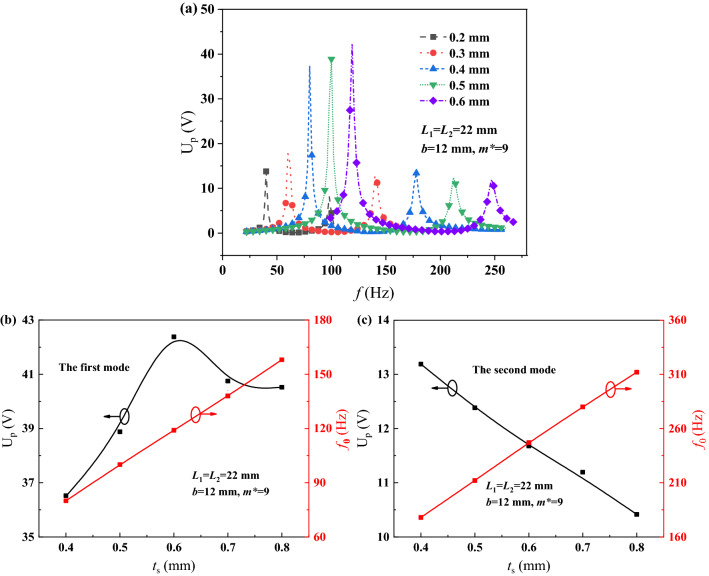
The U_p_-*f* curves (**a**), the U_p-max_ and *f*_0_ vs *t*_*s*_ curves at the first (**b**) and second (**c**) modes of PEHDCB.

The U_p_-*f* curves were simulated under the different substrate thicknesses *t*_*s*_, such as 0.4, 0.5, 0.6, 0.7 and 0.8 mm, and they are described as Fig. 7a, when m^*^ = 9, *b* = 12 mm, *L*_1_ = *L*_2_ = 22 mm. Analogously, the U_p-max_ and *f*_0_ vs *t*_s_ curves are described as Fig. 7b for the first mode and Fig. 7c for the second mode. With the increase of *t*_s_, the U_p-max1_ increases to reach the peak at *t*_*s*_ = 0.6 mm however the U_p-max2_ decreases monotonously. Both the *f*_01_ and *f*_02_ increase. It could guide to choose a suitable *t*_s_ to achieve high U_p-max_ and low *f*_0_ of PEHDCB under the multiple-frequency excitation.

### Multi-factor analysis via orthogonal test method

Generally, the effect of single-factor on performance of PEHDCB could not be taken into account the cross factor effect^33^, therefore we carry out the multi-factors analysis to optimize energy harvesting performance of PEHDCB by using orthogonal test method. The orthogonal factor/level list and the L_25_ (5^6^) corresponding orthogonal list are shown in Tables 3 and 4.

**Table 3:** The orthogonal factor and level list.

Factor	Level				
	1	2	3	4	5
*d* (mm)	2	3	4	5	6
*t*_s_ (mm)	0.4	0.5	0.6	0.7	0.8
*L* (mm)	26	27	28	29	30

**Table 4:** L_25_ (5^6^) orthogonal list.

Cases	Factor			Δ*f*_0_ (Hz)	U_p-max1_ (V)	U_p-max2_ (V)
	*d* (mm)	*t*_s_ (mm)	*L* (mm)			
1	2	0.4	26	104.9	25	14.7
2	2	0.5	27	117.2	33.4	14.2
3	2	0.6	28	128	37.5	14.2
4	2	0.7	29	137.2	34.4	12.5
5	2	0.8	30	145.5	40.4	12.3
6	3	0.4	27	102.7	28.6	14.6
7	3	0.5	28	115	34.4	14.2
8	3	0.6	29	126.1	38.7	13.5
9	3	0.7	30	135.7	41.6	12.4
10	3	0.8	26	163.1	35.6	13.2
11	4	0.4	28	100.7	31.4	14.5
12	4	0.5	29	113.1	38.0	13.7
13	4	0.6	30	124.6	41.7	12.8
14	4	0.7	27	148	37.0	13.1
15	4	0.8	26	134.6	32.7	12.0
16	5	0.4	29	99	34.8	13.9
17	5	0.5	30	111.7	40.0	12.8
18	5	0.6	28	132.6	38.3	13.0
19	5	0.7	27	149.8	37.4	12.7
20	5	0.8	26	167	35.8	12.5
21	6	0.4	30	97.0	37.3	13.3
22	6	0.5	28	118.8	35.5	14.0
23	6	0.6	27	138	36.4	13.8
24	6	0.7	26	156.1	36.2	12.5
25	6	0.8	29	157.1	40.8	10.8

There are the three geometrical dimensions *d*, *t*_s_ and *L* corresponding to 5 levels in Table 3. Distinguished from the traditional orthogonal test method^8^, the U_p-max1_, U_p-max2_ and Δ*f*_0_ were chosen as the optimization objectives to analyze the influences of the three factors on the performance, and the last three factors are kept empty corresponding to the L_25_ (5^6^) lists in Table 4^34^. As for the optimal combination of Case 21 in Table 4, the U_p-max1_ and U_p-max2_ are 37.3 V and 13.3 V, and the Δ*f*_0_ is 97.0 Hz. Correspondingly, the *d, L* and *t*_s_ are optimally determined as 6, 30, and 0.4 mm, in order that PEHDCB achieves not only high U_p-max_ but also narrow Δ*f*_0_ for easily harvesting electrical power from the multiple-frequency excitation^17^.

## Experiment

### Experimental measurement

**Figure 8:**
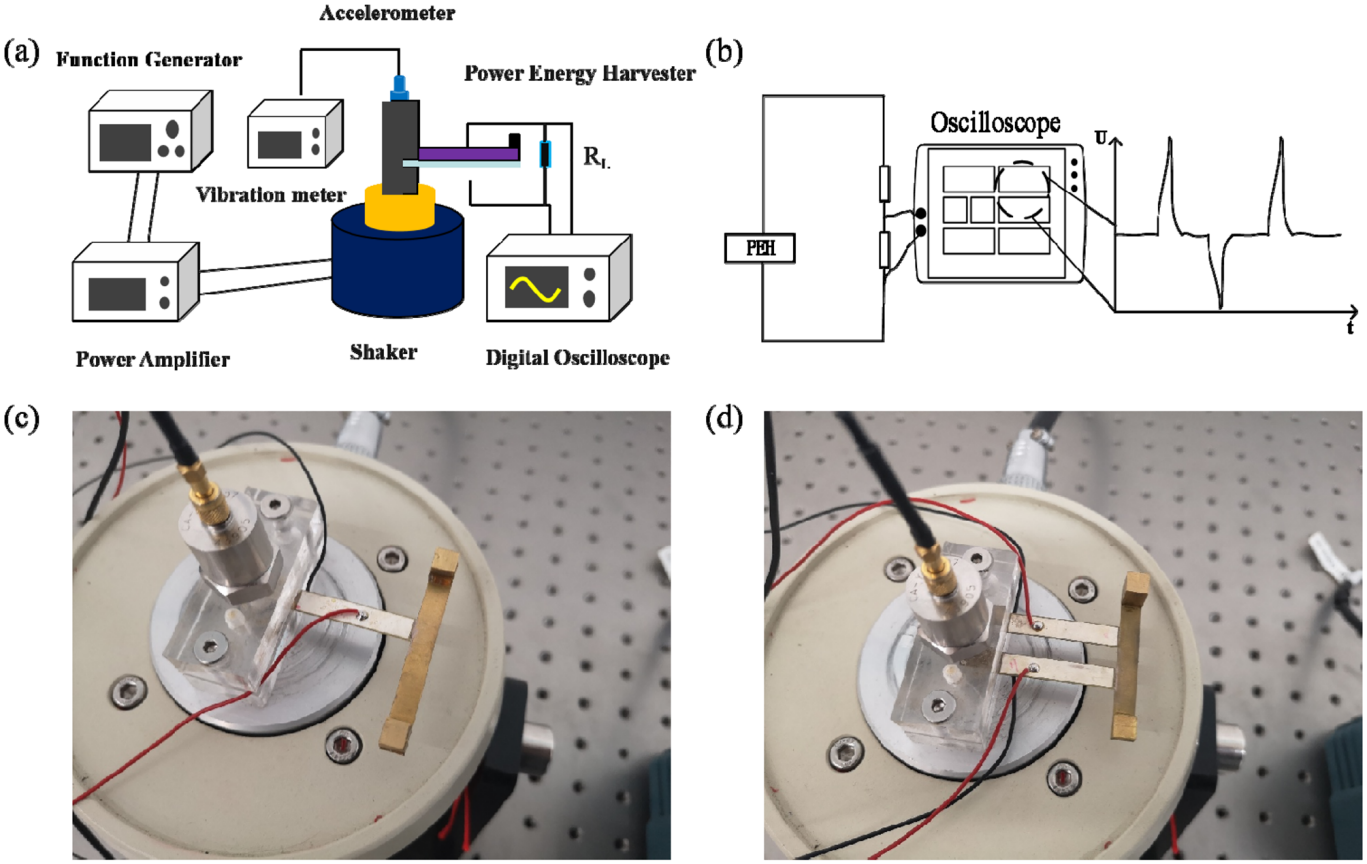
Schematic diagram (**a**). Power measurement circuit (**b**). The vibration component prototypes of PEHSCB (**c**) and PEHDCB (**d**).

The mechanical vibration experiment was carried out to verify the validity and accuracy of simulation result, and the schematic diagram and power measurement circuit are given in Fig. 8a,b for the measurement setup^27^. In order to investigate the effect of width splitting method on the energy harvesting performances, PEHDCB was constructed by splitting equally the primary beam, and both of PEHSCB and PEHDCB were fabricated according to the optimal combination Case 21 in Table 4, as shown in Fig. 8c,d. After clamped by the fixtures, PEHSCB and PEHDCB were fixed on the shaker (JZk-5, Sinocera Piezotronics, Inc., Jiangsu, China), and the acceleration was obtained through the accelerometer (CA-DR-005, Sinocera Piezotronics, Inc., Jiangsu, China) placed on the top of the shaker. The sinusoidal vibration from the function generator (Tektronix AFG 3021B, Tektronix) was amplified by the power amplifier (YE5871A, Sinocera Piezotronics, Inc., Jiangsu, China), and the shaker was applied at 2.1 A and 1 V to generate the mechanical energy.

All the output peak voltages of PEHSCB and PEHDCB were measured at the certain resistance of 100 kΩ by Digital Oscilloscope (Tektronix TDS 1002, Tektronix), and the output power was calculated by using the formula $P = U_{P}^{2} /2R_{L}$ ^24^_,_ where $R_{L}$ the load resistance. Generally, the proportion of half-power bandwidth and the quality factor (Q = *f*_0_/δ_*f*_) are used to evaluate the collection efficiencies of PEHDCB under a single-frequency excitation and multi-frequency excitation^19,21^, and the δ_*f*_ is the 3 dB bandwidth of PEHDCB^35^.

### Test and verify on energy harvesting efficiency of PEHSCBs

**Figure 9:**
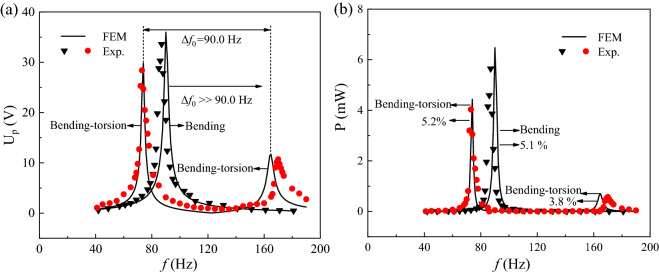
The U_p_-*f* (**a**) and P-*f* (**b**) curves of PEHSCBs undergoing coupled bending-torsion vibrations and bending vibration.

The energy harvesting efficiency of PEHSCB undergoing coupled bending- torsion vibrations is better than that of PEHSCB undergoing bending vibration in the simulation results^24^, therefore we hope to verify the previous results by comparing with the Δ*f*_0_ and proportion of half-power bandwidth in the mechanical vibration experiment as follow. The output peak voltages were measured under *b*** = **12 mm, *L*_1_ = *L*_2_ = 22 mm, R_L_ = 100 kΩ, and the U_p_-*f* and P-*f* curves are given in Fig. 9a,b for PEHSCBs undergoing coupled bending-torsion vibrations with m^*^ = 9 and bending vibration with m^*^ = 1. Here, the simulation results are represented by the solid lines, and the experimental results are described by the dots for the latter and the triangles for the former. From Fig. 9a, the *f*_01_ of PEHSCB with m^*^ = 9 is lower than that of PEHSCB with m^*^ = 1, which indicates that PEH undergoing coupled bending-torsion vibrations is more suitable for harvesting the electrical power from the ambient vibration composed of low frequency^36^. On the other hand, the Δ*f*_0_ is 90.0 Hz for PEHSCB undergoing coupled bending-torsion vibrations, however it is far much narrower than that of PEHSCB undergoing bending vibration. Obviously, the coupled bending-torsion vibrations could decrease the Δ*f*_0_ to allow the harvesting of electrical power from the multiple-frequency excitation, and the results are similar with the experimental observations in the previous reports^17,37^. The energy harvesting efficiency could be generally evaluated by the half-power bandwidth value^19,21^, but the proportion of half-power bandwidth should be more suitable to evaluate the harvesting efficiency for PEH at the multiple-frequency excitation^25^. Here, the proportions of half-power bandwidth are 5.2% at the first mode and 3.8% and at the second mode for PEHSCB with m^*^ = 9, and the proportion of half-power bandwidth is 5.1% at the first mode for PEHSCB with m^*^ = 1. Obviously, the proportion of half-power bandwidth of PEHSCB undergoing coupled bending-torsion vibrations is superior to that of PEHSCB undergoing bending vibration at the first mode, meanwhile the former could far more easy to harvest the electrical power from the ambient vibration because of the resonance lack for the latter at the second mode. The energy harvesting efficiency of PEHSCB undergoing coupled bending-torsion vibrations must be much better than that of PEHSCB undergoing bending vibration, since the environmental vibrations are generally composed of the multiple-frequency.

### Effect of width splitting method on the energy harvesting performances

**Figure 10:**
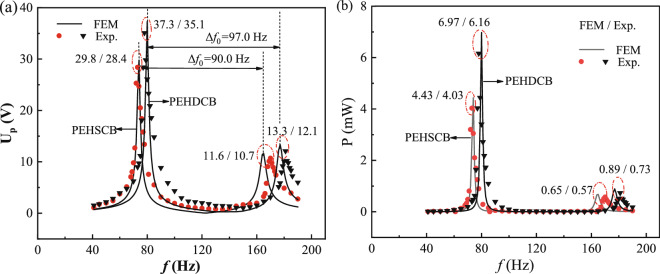
Effect of width splitting method on the U_p_-*f* (**a**) and P-*f* (**b**) curves of PEHs undergoing coupled bending-torsion vibrations.

In order to verify the effect of width splitting method on the energy harvesting performances^19,21^, the U_p-max_ and P_p-max_ of PEHDCB are compared with those of PEHSCB, meanwhile the quality factors related with Δ*f*_0_ are compared because of damping change induced width splitting method. The U_p-max_ were measured for PEHDCB with *d* = 6 mm, *b*** = **12 mm, *L*_1_ = *L*_2_ = 22 mm, m^*^ = 9, *R*_L_ = 100 kΩ, and the U_p_-*f* and P-*f* curves are given in Fig. 10a,b for both of PEHSCB and PEHDCB. The simulation/experimental U_p-max1_ and U_p-max2_ results are 37.3 V/35.1 V and 13.3 V/12.1 V for PEHDCB, and they are 29.8 V /28.4 V and 11.6 V/ 10.7 V for PEHSCB. The P_p-max1_ and P_p-max2_ are 6.97 mW/ 6.16 mW and 0.89 mW/ 0.73 mW for PEHDCB, and they are 4.43 mW/ 4.03 mW and 0.65 mW/ 0.57 mW for PEHSCB. For the same undergoing coupled bending-torsion vibrations, the U_p-max_ and P_p-max_ of PEHDCB are obviously larger than those of PEHSCB, and they are agreement with the simulation reports^19,21^. As for the first and second modes, the simulation/experiment Q_1_ and Q_2_ results are 32.13/25.08 and 35.4/30.17 for PEHDCB, and they are 29.72/24.33 and 33.15/25.2 for PEHSCB. Obviously, the Q_1_ and Q_2_ of PEHDCB undergoing coupled bending-torsion vibrations are larger than those of PEHSCB undergoing bending vibration, and it indicates that the former could easily harvest the energy from the ambient vibration. In a word, all of the energy harvesting performances of PEHDCB undergoing coupled bending-torsion vibrations have been enhanced by the width-splitting method. Our result shows that bending torsional coupled vibration PEH can harvest more power than bending vibration PEH, which is consistent with the result reported^38^.

### Energy harvesting performances of asymmetry PEHDCBs

**Figure 11:**
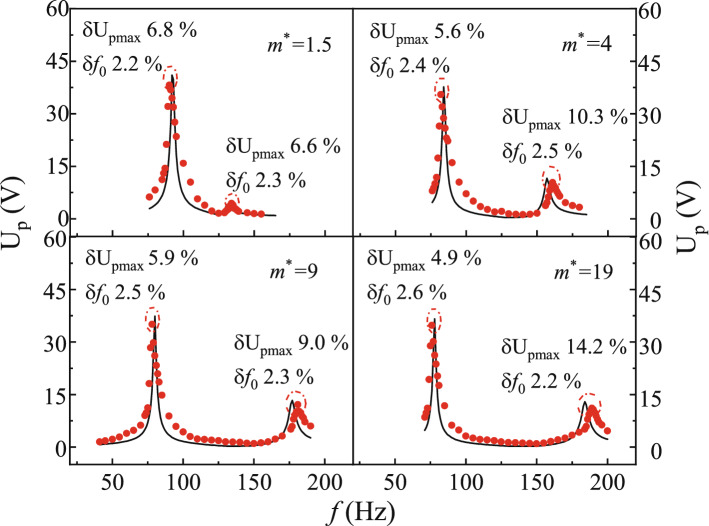
The U_p_-*f* curves under the different mass ratios m^*^ for the PEHDCBs.

The U_p_-*f* curves of the PEHDCBs were measured under the different mass ratios m^*^, such as 1.5, 4, 9 and 19, and they are given in Fig. 11, when *b* = 12 mm, *L*_1_ = *L*_2_ = 22 mm, *d* = 6 mm, R_L_ = 100 kΩ. Here, the simulation and experimental results are represented by the lines and dots, in order that the validity could be confirmed by analyzing the relative deviations of U_p-max_ and *f*_0_ at the first and second modes. Obviously, the maximum relative deviations of U_p-max_ and *f*_0_ are 6.8% and 2.6% for the first mode, and they are 14.2% and 2.5% for the second mode. There are generally the incomplete clamping of the experimental fixture and the unavoidable assembly errors in the manufacture^39^, therefore the simulation results could be not exactly equal to the resonance frequency experiment results for PEHs with the asymmetric mass and width-splitting beam in Figs. 8, 9 and 10. The clamped end of beam is not completely rigid in mechanical vibration experimental, however the fixed boundary conditions in simulation make the beam slightly stiffer. The incomplete clamping and inevitable assembly frequency errors are approximately at the ranges of 2–7% for the nonstable nonlinear energy harvester^40,41^ and 5–10% for the piezoelectric energy harvester for harnessing energy from flow-induced vibration^39^, and the U_p-max_ error is approximately at the range of 10–26% for the rotational mechanical plucking energy harvester^42^. The U_p-max_ and *f*_0_ deviations are 14.2% and 2.6% for the PEHDCBs under the different mass ratios, as shown in Fig. 11, therefore the results are acceptable for the mechanical vibration experiment.

**Figure 12:**
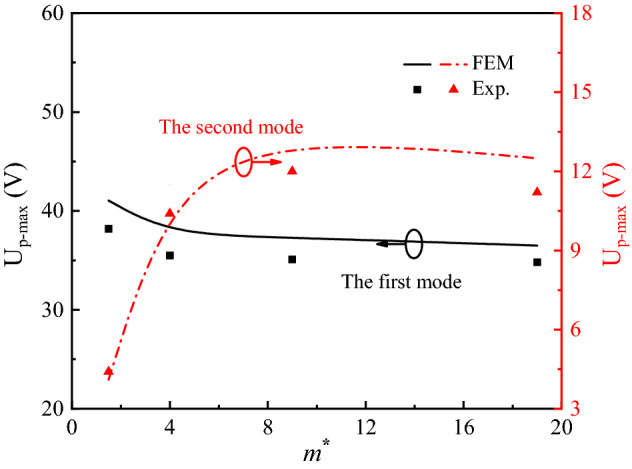
The U_p-max_ vs m^*^ curves at the first and second modes for PEHDCB.

Drawn from Fig. 11, the U_p-max_ and m^*^ are served as the vertical and horizontal ordinates to understand the effect of asymmetric mass on energy harvesting performance, and the U_p-max1_ and U_p-max2_ vs m^*^ curves are described as Fig. 12. With the asymmetric increase of m^*^ from 1.5 to 9, the simulation/experimental U_p-max1_ values decrease from 41.0 V/38.2 V to 36.5 V/34.8 V, and the simulation/experimental U_p-max2_ values increase to the reach peak values of 13.3 V/12.8 V and 12.1 V/11.1 V. Obviously, the energy harvesting efficiency is slightly decreased under the multiple-frequency excitation when the mass ratio m^*^ is larger than 9. Considering the trade-off of U_p-max1_ and U_p-max2_, PEHDCB with m^*^ = 9 can harvest energy more evenly, and it is similar with the reported result^43^. In a word, there is a useful strategy to enhance the U_p-max1_ and U_p-max2_ by adjusting the asymmetric mass ratio under the multiple-frequency excitation. There is an impact of fatigue life on the energy harvest from environmental vibration with a multiple-frequency excitation, and the fatigue life could be chosen as the indicative parameters at the multi-factors analysis the further work including experiment and simulation.

## Conclusion

In summary, the optimization geometrical parameters of PEHDCB are determined by considering the U_p-max_ and Δ*f*_0_ in the multi-factor analysis based on the orthogonal test, and the Δ*f*_0_ narrowed of PEHDCB undergoing coupled bending-torsion vibrations results in harvesting the more electrical power from the ambient vibration composed of low frequency. The quality factors Q_1_ and Q_2_ of PEHDCB undergoing coupled bending-torsion vibrations are larger than those of PEHSCB under the multi-frequency excitation, and the U_p-max1_ and P_p-max1_ at the first mode are increased 25.2% and 57.3% for PEHDCB. The width-splitting method could be successfully used to improve the energy harvesting performances, and the measurement reliability is acceptable considering the incomplete clamping, damping and inevitable assembly effects. Considering the trade-off of U_p-max1_ and U_p-max2_, the asymmetry PEHDCB with the mass ratio determined as 9 can harvest energy more evenly, and the results are effective to predict the energy harvesting performances for PEHDCB undergoing coupled bending-torsion vibrations under the multi-frequency excitation. As for the purpose to explore the effect of width splitting method on the energy harvesting performances in this work, we should consider the parameters related with damping change such as the width splitting size *d* into electromechanical coupling dynamic equation, and one could obtain a closed form solution and comparing to give better insights on how the harvested power varies by varying the parameters in future research.
